# Association of Diopsys® Short-duration Transient Visual Evoked Potential Latency with Visual Field Progression in Chronic Glaucoma

**DOI:** 10.5005/jp-journals-10028-1240

**Published:** 2018-03-01

**Authors:** Richard Trevino, William E Sponsel, Carolyn E Majcher, Joey Allen, Jeffery Rabin

**Affiliations:** 1 Optometrist, Rosenberg School of Optometry, University of the Incarnate Word, San Antonio, Texas, USA; 2 Director, Professor and Consultant, Department of Ophthalmology, CLI Eyes of Africa Clinic and Surgery Center, Malawi, Africa; WESMDPA Baptist Medical Center Glaucoma Service, San Antonio, Texas; Department of Biomedical Engineering, University of Texas San Antonio, San Antonio, Texas; Rosenberg School of Optometry, University of the Incarnate Word, San Antonio, Texas, USA; 3 Optometrist, Rosenberg School of Optometry, University of the Incarnate Word, San Antonio, Texas, USA; 4 Optometrist, Rosenberg School of Optometry, University of the Incarnate Word, San Antonio, Texas, USA; 5 Optometrist, Rosenberg School of Optometry, University of the Incarnate Word, San Antonio, Texas, USA

**Keywords:** Cohort study, Electrophysiology, Glaucoma, Perimetry, Visual evoked potential.

## Abstract

**Aim:**

To determine the association of Diopsys® NOVA-LX amplitude and latency abnormality scores with perimetric staging of chronic glaucoma, and to explore potential single-visit short-duration transient visual evoked potential (SD-tVEP) trend detection ability utilizing Humphrey 30-2 field progression data.

**Materials and methods:**

*Setting: *Glaucoma subspecialty clinic. *Participants: *Treated adult chronic glaucoma patients undergoing SD-tVEP evaluation. *Main outcome measures: *(1) Proportion of eyes designated as suspect or abnormal by the NOVA-LX multifactorial algorithm were determined as a function of glaucoma severity using the most recent Humphrey visual field analyzer (HVFA) 30-2 field. (2) Association between long-term HVFA-guided progression analysis (GPA) annual slopes and SD-tVEP abnormality was assessed to determine whether a single VEP test might help to identify eyes more prone to progressive visual field (VF) loss.

**Results:**

One hundred and thirty-three eyes of 84 patients (mean age 68 years) were analyzed. The SD-tVEP abnormality increased proportionately with severity of VF loss under high-contrast (Hc) test conditions for both latency (p = 0.001) and amplitude (p < 0.01). The HVFA progression analysis printouts existed for 91 eyes (mean 12.3 fields per eye/range 5-18). Nearly three-quarters (72.5%) of eyes with mean annual HVFA progression >0.7 dB/year (n = 29) had single-visit VEP latency abnormalities. Fewer than half (46.7%) of the remainder (n = 62) showed latency abnormality. Mean progression for eyes with abnormal *vs *normal VEP latency was -0.87 ± 0.3 dB/year *vs *-0.32 ± 0.4 dB/year.

**Conclusion:**

Diopsys NOVA-LX Hc latency abnormality shows strong association with VF loss among a diverse population of clinical patients undergoing active treatment for chronic glaucoma, and appears likely to afford clinically useful trend-detecting test.

**Clinical significance:**

The SD-tVEP has the potential to serve as a single-visit clinical indicator to identify glaucoma patients at high risk for VF progression.

**How to cite this article: **Trevino R, Sponsel WE, Majcher CE, Allen J, Rabin J. Association of Diopsys® Short-duration Transient Visual Evoked Potential Latency with Visual Field Progression in Chronic Glaucoma. J Curr Glaucoma Pract 2018;12(1):29-35.

## INTRODUCTION

The VEP is an objective measure of visual function that assesses the electrical activity of the cerebral cortex using electrodes placed on the scalp, while the subject views standardized visual stimuli.^1,2^ The amplitude and latency of the VEP waveforms are affected by various pathologic conditions of the visual pathway,^3^ including glaucoma.^4-6^ Towle et al^7^ found that increased pattern VEP latency was associated with severity and location of glaucomatous VF defects, the extent of optic nerve cupping, and disk pallor. They also showed VEP latency prolongation was frequently abnormal in ocular hypertensives but not in control eyes. Recent technological developments have made VEP assessment faster, more reproducible, with interpretation of test results more objective, and have brought down the cost of the instrumentation. One such technological innovation is SD-tVEP. This process decreases test duration substantially by means of synchronized signal acquisition in combination with a postprocessing technique that provides less subjectivity in waveform assessment.^8^

Pillai et al^9^ have recently reported on the ability of SD-tVEP latency to objectively discriminate between normal and glaucomatous eyes. In this study, we endeavored to assess rates of abnormal SD-tVEP amplitude and latency findings in adults under therapy for chronic glaucoma using the Diopsys Nova-LX P100/N75-referenced Hc and low-contrast (Lc) stimuli, with the ultimate goal of determining the potential clinical value of single-visit SD-tVEP as a potential indicator of VF progression risk. Low-contrast testing demonstrates degradation of magnocellular pathways, and might be expected to help provide an early indication of glaucoma. High-contrast testing demonstrates degradation of parvocellular pathways and might be anticipated in glaucomatous eyes with or at risk for pericentral visual loss. Any dynamic neural function test able to demonstrate strong association with the presence of disease might have potential to help distinguish adequately treated eyes that are likely to remain stable from those that may progress despite apparently good intraocular pressure (IOP) control. A measure of signal intensity, VEP amplitude, should be reduced by both the absence of lost axons and inactivity of extant but dysfunctional axons. Conversely, VEP latency, a measure of conduction velocity, can only be affected by the state of intact neural pathways, since dead axons send no contributory signal to the occipital cortex. Thus, it is reasonable to anticipate that among a diverse clinical population receiving aggressive therapy, eyes with normal VEP latencies might be more likely to remain clinically stable and at lower risk of continued axonal loss, while those with significant latency delay might be more likely to continue to lose even more of their already depleted complement of axons. If so, appropriate adjustments of therapeutic intervention and follow-up rates for higher- and lower-risk patient subpopulations could decrease visual morbidity and save considerable time and expense. Visual fields and optical coherence tomography are great clinical assets for helping discern which eyes exhibit structural and functional damage, but neither modality can readily provide evidence based on single visit on which eyes might be more or less prone to progression until multiple studies have been performed. It may require 24 to 36 months of periodic follow-up assessment to verify mild-to-moderate VF progression or nerve fiber layer loss, during which time the adequacy of ongoing therapy is uncertain, and potentially preventable permanent axonal loss is allowed to progress.

Approximately 2000 Diopsys units are in present use in the United States and Canada (Matthew Emmer, Diopsys Inc.; personal communication). These devices utilize a standardized protocol outlined previously.^5^ Reliability and pathologic indices are automatically assessed by the device, in analogous manner to those generated by familiar tomographic and perimetric devices in widespread clinical use. Stimulus luminance, contrast, size, electrode placement, filter settings, and the normative database employed by the system are all standardized. Quantitative numerical amplitude or latency values are used to generate pathological indices using a proprietary software algorithm. A reliability index is generated based on waveform structure and artifact detection by the software system.

The objective of this study is to evaluate the potential clinical utility of this commercially available test as administered by trained ophthalmic technicians under manufacturer recommended conditions using the current version of the standardized NOVA-LX semi-automated protocol in the evaluation and monitoring of patients with chronic glaucoma. It is important to emphasize that this is not an electrophysiological study intended to explore the strengths and weaknesses of SD-tVEP methodology. It is a strictly practical assessment of a new and now widely used commercial product simplified for practicing clinicians very familiar with the intellectual challenges of integrating multiple imperfectly measured physiologic risk factors into their decision-making for patients with glaucoma.

## MATERIALS AND METHODS

This cross-sectional protocol was provided an official noninvasive study waiver by the Institutional Review Board of Baptist Health System and the Institutional Review Board of the University of the Incarnate Word, and adhered to the tenets of the Declaration of Helsinki. All data were managed in accordance with the regulations set forth in the Health Insurance Portability and Accountability Act. Clinical records of adults with chronic glaucoma who underwent SD-tVEP evaluation with the Diopsys NOVA LX^®^ system between August 2013 and April 2014 at a single ophthalmology glaucoma specialty practice (WESMD Professional Association) were reviewed. All examinations were performed using a fixed protocol in the same clinical setting by qualified ophthalmic technicians who underwent formal training with the system but with no prior experience in electro-physiologic testing. All eyes had been undergoing therapy with what was deemed to be appropriately aggressive therapy (medical, laser, and/or incisional surgery), and IOPs were thus generally well controlled among all the eyes evaluated in this study (5 mm Hg < IOP < 24 mm Hg; mean ~15 mm Hg).

Details regarding the Diopsys VEP protocol have been published elsewhere,^5^ and are briefly summarized here. The subject is positioned 1 meter from the 17 inch video monitor. All testing is performed monocularly with the subject corrected for the 1 meter working distance. The Diopsys NOVA-VEP uses a three-electrode setup. The active electrode is attached to the scalp at 0z (over the occipital cortex), the reference electrode is placed on Fz (on the center of the patient’s forehead), and the ground electrode is placed on Fp1 (on one side of the patient’s forehead just above the temple as close to the hairline as possible). The stimulus is a 32 × 32 checkerboard pattern that reverses at a rate of 1 Hz (one reversal every 0.5 seconds). The examination sequence consists of an 8-second warm-up period followed by 15 seconds with a 10% contrast (Lc) target, and finally 15 seconds with an 85% contrast (Hc) target. Each stage of the examination may be repeated if the patient was not attending to the stimulus during the examination. The fellow eye is then tested in an identical manner.

For each eye, after the amplitudes and latencies of the SD-tVEP under both Lc and Hc stimuli conditions were collected, the VEP findings were interpreted by the Diopsys device’s intrinsic first-generation software as being normal, suspect, or abnormal. For patients with more than one VEP examination in their record, data from the exam in closest proximity to their most recent VF was collected. The mean deviation (MD) of the most recent Humphrey 30-2 SITA-standard VF was then used to stage the severity of the glaucoma as being mild (MD > -6.00 dB), moderate (-6.00 dB ≥ MD > -12.00 dB), or severe (MD ≤ -12.00 dB). Finally, the progression of VF change over the preceding 3 to 7 years for each subject was determined using HVFA intrinsic GPA software on all subjects who had previously undergone at least five reliable VFs that generated an automated printout. The mean Visual Function Index percentage VF survival status for each eye was recorded as the midpoint value for the established linear progression.

For inclusion in the study, patients were required to be over 18 years of age at the time of their examination and carry a diagnosis of chronic glaucoma. Patients with all forms of chronic glaucoma were eligible for inclusion, including open-angle, narrow-angle, stabilized uveitic, posttraumatic, and congenital glaucoma. Because we are not aware of any reported differences in electrophysi-ologic defects occurring among the various subtypes of chronic glaucoma, we included patients with all forms of chronic glaucoma in this analysis. Patients with ocular comorbidities that could confound VF interpretation, such as dense cataract or advanced maculopathy were excluded. Only clinical records containing a complete data set were included in the final analysis. A complete data set consisted of (1) SD-tVEP examination using both Lc and Hc targets each containing a P100 wave that was identified and interpreted by the device as normal or abnormal; and (2) a reliable Humphrey 30-2 automated perimetry examination (false positive, false negative, and fixation loss all <25%) performed within 6 months of the VEP examination.

The unit of analysis for this study was each eye of a patient that met the inclusion criteria. Both eyes of a patient with bilateral glaucoma were included if they both met the inclusion criteria. Amplitude and latency data were tabulated for each eye. Any interpretation other than ‘normal’ for each parameter was categorized as ‘abnormal.’ This included both red (outside normal limits) and yellow (borderline) coded results in the Diopsys VEP histogram report. For the comparisons of latency with VF progression, the absence of a numerical latency value in eyes capable of generating a waveform acceptable for automated amplitude analysis was also deemed to be an abnormal latency result. The MD of the HVFA automated perimetry exam performed at or closest to the date of the VEP examination was recorded and used to stage the severity of the glaucoma as follows—mild: MD > -6.00 dB; moderate: -6.00 dB ≥ MD > -12.00 dB; severe: MD ≤ -12.00 dB. Probability values are reported for differences attaining statistical significance <0.05. Single-factor analysis of variance was used to test for VEP differences between staged groups, and two-tailed t-tests were used to identify differences between the Hc and Lc amplitude and latency testing responses. The McNemar chi-square test was used to compare rates of abnormality of the P100 latency and amplitude under Lc and Hc test conditions within each staged group. Mantel-Haenszel linear-by-linear association analysis was used to test for trend in abnormality rates across staged groups. All statistical analyses were performed using Statistical Package for the Social Sciences Statistics version 21 (IBM Corporation, Armonk, New York, USA).

## RESULTS

A total of 88 patients underwent SD-tVEP examination during the study period (38 males, 50 females, mean age: 68 years). Of these, four were excluded because complete data were not available for either eye. Of the remaining 84 patients (37 males, 47 females, mean age: 68.6 ± 12.9 years, range: 23-91 years), 49 (58%) had complete data available for both eyes and 35 (42%) had complete data for one eye only. Of the 35 patients with data available for one eye only, 28 (80%) had only one eye with sufficient visual function to undergo testing. The remaining 7 (20%) had undergone testing of both eyes but the Diopsys device was unable to interpret either the amplitude or latency of the waveform under Hc, Lc, or both test conditions in the fellow eye. Hence, our total study sample consisted of 133 eyes (Flow Chart 1). Of these, 71 eyes (53%) were categorized as having mild VF loss (MD < -6 dB), 20 eyes (15%) had moderate loss (MD: -6 to -11.99 dB), and 42 eyes (32%) had severe loss (MD ≥ -12 dB). Mean age was 68.2 ± 1.3 and did not differ significantly between glaucoma conditions (F = 0.85, p > 0.43). Visual field progression data were available for all these eyes. Table 1 summarizes the number of eyes with a VEP abnormality in each staged group.

**Flow Chart 1:**
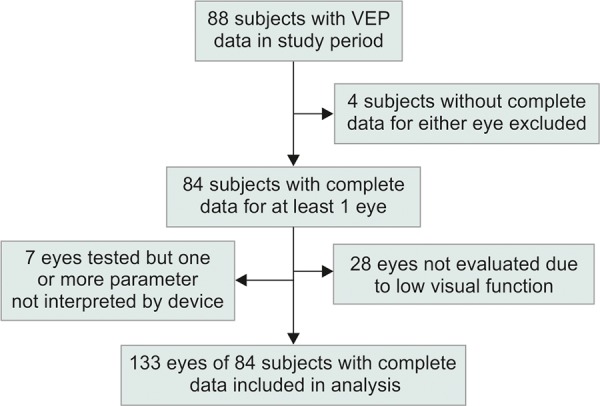
Subject exclusion

**Table 1:** **Table 1: **SD-tVEP abnormality by staged group*

		*n*		*MD^†^*		*LCA*		*LCL*		*HCA*		*HCL*	
Mild		71		–1.19 ± 2.49		6 (8.5%)		10 (14.1%)		4 (5.6%)		16 (22.5%)	
Moderate		20		–9.12 ± 1.53		1 (5.0%)		5 (25.0%)		0 (0.0%)		8 (40.0%)	
Severe		42		–19.77 ± 4.58		3 (7.1%)		12 (28.6%)		10 (23.8%)		22 (52.4%)	

*Expressed as number of eyes with specified abnormality (%); ^†^mean ± standard deviation; Mild: visual field mean deviation of < -6 dB, Moderate: visual field mean deviation between -6 and -11.99 dB, Severe: visual field mean deviation of ≥ -12 dB; MD: Mean deviation; LCA: Low-contrast amplitude; LCL: Low-contrast latency, HCA: High-contrast amplitude; HCL: High-contrast latency

Among the 71 eyes with mild VF loss (mean MD: -1.19 dB), latency abnormalities occurred more frequently than amplitude abnormalities. This difference was not statistically significant under Lc conditions, but achieved statistical significance under Hc conditions (p < 0.01). The VEP abnormalities occurred more often using Hc targets than Lc targets, but this difference did not achieve statistical significance for either amplitude or latency parameters.

Of the 20 eyes with moderate VF loss (mean MD: -9.12 dB), none demonstrated an amplitude abnormality under Hc test conditions, and only one had an amplitude abnormality under Lc conditions. The absence of any Hc amplitude abnormalities in this group prevented us from performing McNemar analysis on this VEP parameter. There was no significant difference in the prevalence of other VEP parameter abnormalities (Lc amplitude *vs *Lc latency and Hc latency *vs *Lc latency).

Among the 42 eyes with severe VF loss (mean MD: -19.77), VEP abnormalities occurred significantly more often using Hc targets than Lc targets (latency: p = 0.02, amplitude: p = 0.04), and latency defects were significantly more common than amplitude defects under both Lc and Hc conditions (Lc: p = 0.04, Hc: p < 0.01).

**Graph 1:**
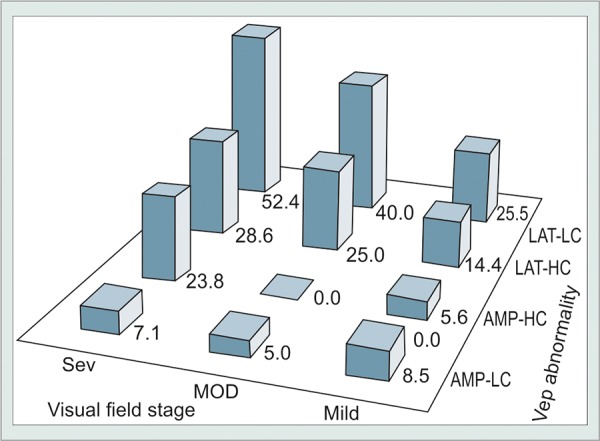
Rate of SD-tVEP abnormality by staged severity group. Bars represent the proportion of eyes scored as abnormal by the Diopsys Nova-LX automated analysis software for Lc and Hc SD-tVEP amplitudes (AMP) and latencies (LAT) in actively treated glaucomatous eyes demonstrating mild (MILD), moderate (MOD), or severe (SEV) Humphrey 30-2 visual field survival status. Note that amplitude would be expected to reflect the combined extent of axonal dysfunction and axonal loss, while latency would be expected to reflect the conduction velocity along surviving and functionally active axonal pathways. Glaucomatous eyes with less VF pathology demonstrated significantly lower rates of SD-tVEP latency failure. Eyes with normal latency values also tended to demonstrate lower rates of ongoing VF progression than those with abnormal latency values among the total study population (see results)

There was a clear trend of greater prevalence of VEP abnormality using the Diopsys scoring algorithm with increasing severity of VF loss (Graph 1). This association achieved statistical significance under Hc test conditions (Hc latency: p = 0.001; Hc amplitude: p < 0.01) and approached significance for Lc latency (p = 0.06).

In view of the relative strength of these associations of latency abnormality with VF status, latency abnormality (Hc and/or Lc) was the comparative indicator we then used to explore the most recent HVFA GPA printouts among our study population for any potential association between VF progression and SD-tVEP (Table 2). Ninety-one of the 133 eyes (68%) had the minimum requirement of five VFs required to generate a GPA linear regression slope, and eyes accorded significant progression status typically demonstrated annual loss in MD of worse than 0.7 decibels (dB). The GPA compilation comprised nearly 1,200 VFs (mean 12.2 ± 0.4 VFs per evaluated eye; range 5-18). The mean mid-slope GPA Visual Field Index percentage VF survival status among the 91 eyes was 68.8 ± 2.8% across a full function range from 0 to 100%. The mean overall progression rate was -0.57 ± 0.22 dB/ year. Eyes demonstrating any VEP latency abnormality produced a composite GPA slope of -0.87 ± 0.3 dB/ year, while those with all-normal VEP latency results produced a composite GPA slope of -0.32 ± 0.4 dB/year. Twenty-nine eyes (31.9%) had severely progressive GPA slopes ≥ -0.7 dB/year, among which 21 (72.5%) showed VEP latency abnormality (Hc and/or Lc). Sixty-two eyes (68.1%) had less severe or positive GPA slopes < -0.7 dB/year, among which 29 (46.7%) showed VEP latency abnormality.

**Table 2:** **Table 2: **Rate of disease progression by SD-tVEP latency abnormality*

*GPA slope*		*Abnormal latency*		*Normal latency*		*Total*	
≥ 0.7 dB/year		21 (72.4%)		8 (27.6%)		29	
< 0.7 dB/year		29 (46.8%)		33 (53.2%)		62	
Mean^†^		–0.87 ± 0.3		–0.32 ± 0.4			

*Expressed as number of eyes (%); ^†^Mean ± standard deviation

## DISCUSSION

The glaucomas, collectively, are a composite of ocular neurodegenerative conditions with the common features of axonal and VF loss. Historically, associations between clinically measured risk variables (IOP, optic disk cupping, VF loss) have been surprisingly weak.^10-13^ No single test can detect all glaucoma, in part because of the diverse character of the many pathologic subtypes that exist. Observable relationships between VF loss and nerve fiber loss have become much clearer over the past two decades as computerized perimetry and tomographic methods have simultaneously advanced. Indeed, such refinements have helped confirm the coordinating role played by the central nervous system in the bilateral progression of chronic glaucoma.^14^ Future therapeutic advances will require a further delineation of glaucoma-tous subtypes, based on traditional clinical evaluation alongside complimentary genetic and neurophysiologic findings. One key to this etiologic delineation will be to determine whether a particular individual’s visual deficit is a consequence of intraretinal dysfunction, visual pathway compromise, or both. While both VEP and electroretinogram (ERG) can now be conducted with the Diopsys system, at the time of this study only the SD-tVEP modality was conducted, which provided a standardized intrinsic scoring algorithm allowing for objective clinical comparison with VF status. The newly introduced, similarly standardized scoring algorithm for the Diopsys ERG modality should facilitate future clinical characterization of etiologic subtypes of chronic glaucoma based on both electrophysiologic modalities.

In this study of VEP, we found that both Lc and Hc latency abnormalities were associated with VF mean defect, but SD-tVEP Hc latency abnormality was consistently and significantly more preponderant in each VF scoring category than Lc latency abnormality. Indeed, Hc latency abnormality was more prevalent than Lc latency abnormality at all severity stages using this instrument’s proprietary algorithm among our study population. Our latency results are comparable to those of Prata et al^5^ who found greater SD-tVEP latency in individual subjects in the eye with greater field loss. Pillai et al^9^ also found a significant correlation between SD-tVEP latency and severity of glaucomatous VF loss, but among their population Lc latency was more strongly correlated with perimetric staging of severity, which may reflect differences in patient populations including glaucoma type, age, gender, and/or racial differences. Recently, Kothari et al^15^ utilized a conventional Hc VEP system and showed significant correlations between both Hc latency and amplitude and VF pattern standard deviation. Giving full respect to all these studies, we chose to characterize any latency failure, Lc or Hc, as the basis for comparison with VF progression among our study cohort to provide a simple primary algorithm that might be generally clinically applicable.

In contrast to latency, we found that SD-tVEP amplitude abnormality occurred relatively infrequently, even in patients with severe VF loss. These results contrast with the findings of Prata et al^5^ who used a similar system to compare fellow eyes of patients with asymmetric glaucoma and found that SD-tVEP amplitude was decreased in eyes with greater glaucomatous VF loss. This may be because their comparisons were within subjects, eliminating the great variability that exists between subjects.^1^ Earlier studies using conventional VEPs also reported correlations between glaucoma severity and VEP ampli-tude.^16^ Because VEP test results are specific to stimulus characteristics, such as check size, reversal rate, as well as display luminance and contrast, differences in test conditions may account for some of these observed differences. The pass/fail criteria for Diopsys SD-tVEP amplitude for glaucomatous eyes under active therapy may need to be refined, but the fact that a very high proportion of eyes produced amplitude values sufficiently robust to allow for automated latency analyses may be considered an advantage in the present context.

Given the very recent introduction of this patient-friendly electrophysiologic technology into the clinical setting, the associations observed between SD-tVEP latency and VF status compare favorably with the associations seen at a similar developmental stage with automated perimetry and nerve fiber layer analysis.^17^ As mentioned previously, unlike amplitude, latency has the potential to help monitor the functional well-being of the surviving functional axons whether they be plentiful or depleted in number. The incremental but strongly proportionate association of latency deficit with VF status among these actively treated eyes thus suggests that our ability to therapeutically normalize axonal conduction rates was directly related to the severity of the disease. This relationship was reflected further by comparisons with long-term VF progression, which indicate that SD-tVEP may afford incremental value as an elective single-visit trend-detection test of dynamic neural function.

## CONCLUSION

In conclusion, Diopsys NOVA-LX® SD-tVEP amplitude and latency abnormalities were significantly associated with VF staging under Hc stimulus conditions. The presence of an SD-tVEP latency deficit was associated with long-term VF progression. The rapidity and clinical ease of the NOVA-LX protocol already allows for more immediate and widespread application of electrophysiology in the clinical realm, but its full potential as a diagnostic, monitoring, and trend-detecting tool for glaucoma management can only be fully realized with continued detailed study and refinement by multiple investigators, in much the same tradition as automated perimetry and computerized tomography.

## CLINICAL SIGNIFICANCE

What is the actual clinical value of this kind of incremental informational yield? Considered in context of disk hemorrhage, the only well-established single-visit clinical indicator predictive of VF loss,^18-24^ the informational yield of this first-generation Diopsys SD-tVEP automated analysis system is noteworthy. De Moraes et al^23^ recently confirmed disk hemorrhage as ‘the single most significant predictor for VF progression.’ Over an 8-year follow-up, Medeiros et al^22^ determined the relative rates for VF progression for eyes with and without disk hemorrhages was -0.88%/year *vs *-0.38%/year, a ratio remarkably similar to those reported here for VEP latency abnormality. The key difference is that disk hemorrhages in Medeiros’ meticulous study arose spontaneously in only 2% of glaucomatous eyes per year. On the contrary, SD-tVEP may be performed electively in the clinic on almost any glaucoma patient at any time.
